# People who inject drugs (PWID) and HIV/aids cases in Mexico City: 1987–2015

**DOI:** 10.1186/s13011-019-0246-x

**Published:** 2019-12-23

**Authors:** Martha Romero Mendoza, Denize Meza-Mercado, Rosario Martínez-Martínez, Carlos Magis-Rodríguez, Arturo Ortiz Castro, Maria Elena Medina-Mora

**Affiliations:** 1 National Institute of Psychiatry, Calzada México-Xochimilco 101, San Lorenzo Huipulco, Ciudad de Mexico, 14370 México; 2 National Program on AIDS (CENSIDA), Homero 213, Polanco, Ciudad de Mexico, 11570 México

**Keywords:** Drug use, Surveillance systems, People who inject drugs, HIV/aids, Mexico City

## Abstract

**Background:**

The purpose of this study is to describe the characteristics of individuals who inject drugs, to explore use trends in the past 25 years, and to review the profile of users of various drugs, both legal and illegal, that have been used intravenously without medical prescription in Mexico City.

**Methods:**

Information was drawn from the Drug Information Reporting System (SRID, 1987–2015) and data from the National Center for the Prevention and Control of HIV/aids (CENSIDA, 1983–2018). SRID is based on two 30-day cross-sectional evaluations carried out during June and November. It has served as an uninterrupted epidemiological surveillance system for 32 years, operating both in health and justice institutions in Mexico City and the metropolitan area. The timely identification of changes in use patterns is regarded as the most consistent tool to track the trajectory of the phenomenon. CENSIDA cases were analyzed based on the number of HIV and aids positive injectable drug users during the same period of time in Mexico City.

**Results:**

Cocaine users represented the highest number of cases, with a total of *N* = 293. Back in 2000, the use of this substance showed a significant increase of up to 50%. In turn, heroine and opiates user trends by sex increased from being almost non-existent in 1987 to 13% in 1994. Results provide evidence of the changes in the number of users over the years, with the largest increases being recorded in 1996 (16.5%), 1999 (17%), and 2010 (13%).

**Conclusions:**

The increase observed in the results coincides with domestic and world political situations that have caused an upturn in the use of some substances over the years. It is not far-fetched to think that in the coming years there will be an increase in the number of individuals who inject drugs due to the high production and availability of heroin in Mexico and the emergence of fentanyl use as indicated by ethnographic research in Mexico City and the deaths linked to its consumption. The latest reports, published in 2018, documented at least five cases of fentanyl users.

## Background

According to various reports by the United Nations Office on Drugs and Crime [1], in recent years there has been a growing concern about the use of injectable substances as there are now eleven million of them the world over. Although this scenario has shown considerable increases in use in countries such as Spain, Holland, Canada, the United Kingdom, and Pakistan, the countries which concentrate 43% of users are China, the United States, and the Russian Federation.

Mexico is a producer and exporter country of some injectable substances, such as heroin, and so an increase in the use among its population [2] and an increase in the cases of HIV and aids are to be expected because several research projects have linked people who inject drugs (PWID) with the risk of contagion [3, 4]. HIV prevalence worldwide among PWID is around 19% [5].

Mexico City is the federal capital of the country and its largest urban area. It is the biggest city in North America with an estimated population of 8.7 million inhabitants, and an estimated 20 million in its metropolitan area, making it the second largest urban conglomerate in the world after Tokyo, Japan. The city has the shape of a large ellipse and an area of 1490 km^2^ [6]. What are the characteristics of PWID living in such a complex megalopolis? What are their substances of choice?

Since illicit drug use is by definition a prohibited behavior, users often wish to be invisible to easily escape detection. Likewise, as drug use is clandestine, it is difficult to design studies to approach users through scientific methods involving the randomized sampling of traditional surveys [7]. Moreover, injectable drug use is an even more socially rejected and stigmatized practice than the use of other psychoactive substances.

Given the above, the study of drug use has relied on a wide variety of methodological alternatives, such as anthropological studies, national addiction surveys, and case reports in institutions that afterwards become registration systems for people who use psychoactive substances and end up at health centers or law enforcement agencies [8].

However, for various reasons, it is important to have a systematic means of monitoring trends in the illicit use of injectable drugs. First of all, it would be desirable conducted on its importance and, when necessary, to implement preventive responses such as education for the community and/or users about possible harms, the reorientation of policy priorities to interrupt the supply of dangerous drugs, the enhancement of services, and the quality of treatment for users with associated problems. Second, data on use and harm-related use are needed to evaluate the effectiveness of policies with the objective of reducing harm; these should include restricting supply and reducing demand for these drugs [8]. Third, this provides a better understanding of the impact caused by the use of psychoactive substances in society, and highlights the gaps between care needs and the supply of services available in the country [9]. Fourth, monitoring systems can be used to visualize changes over time and compare geographic zones, determine priority areas for intervention and better understand the variables of different populations with high drug use rates [10]. Lastly, information systems can potentially reduce health costs, as was the case in this group of injecting drug users, by creating interventions to reduce the spread of HIV, hepatitis, and tuberculosis, and improve the treatment of all of them [11].

The purpose of this study is to describe the characteristics of the cases, use trends, and the profile of users of various drugs, both legal and illegal, that have been used intravenously without medical prescription. They were all collected through the Reporting System and Information of Drugs (SRID 1087–205). It also seeks to track use trends in the past 25 years.

SRID is based on two 30-day cross-sectional evaluations (June and November). It has served as an uninterrupted epidemiological surveillance system for 32 years, operating in health and justice institutions in Mexico City and the metropolitan area. The timely identification of changes in use patterns, together with the recording of trends in various substances, is regarded as the most consistent tool to track the trajectory of the phenomenon [11].

Moreover, data from the National Center for the Prevention and Control of HIV/AIDS-CENSIDA (1983–2018) is analyzed, based on the number of HIV and AIDS positive injectable drug users, during the same period in Mexico City [12].

## Methods

The study is a secondary analysis of the SRID and CENSIDA databases. It is a descriptive, longitudinal, and non-experimental study of the cases. The study is a non-probabilistic sampling. It is a technique where samples are collected through a process which does not offer the same opportunities of being selected to all the members of the population. In this particular situation, the sample is made up of injectable drugs users recorded during the last 25 years [13]. This type of study implies, on the one hand, that there is no sampling framework and so the size and limits of the population are unknown; on the other, there is concern about confidentiality as individuals might belong to stigmatized groups or for having been involved in illegal behaviors [14].

In order to assess injectable drug use, data obtained through the application of the “Individual Report on Drug Use” form during the period 1987–2015 were analyzed.

The SRID sample comprised 740 injectable drug users, who accounted for 1.5% of the total population of first-time visitors (*N* = 46,321 cases) to the participating institutions in the period 1987–2015 and admitted having used intravenous drugs at least once in their lives.

The SRID considers a case any individual who claims having used any non-prescription drug at least once in his/herself lifetime with the deliberate intention of intoxicating his/herself, that is to say, not with medical ends, but for evasion, distraction, or recreational purposes.

Mexico City’s data bases on Epidemiological Surveillance from Ministry of Health’s cases of HIV/aids were also analyzed.

### Instruments

Data used in this study derive from the application of two instruments: first, the Cédula de Registro de Información en Drogas (SRID, Drugs Information Register Schedule) and the second, the Cases Report on HIV/aids of the Direction of Epidemiological Surveillance.

Data used in this study were collected through the application of two instruments.

### SRID drug use variables

Identification data*:* folio, institution, date, name of interviewer, file number, and application of this form at another institution in the past 30 days.

#### Sociodemographic characteristics

Sex, occupation, schooling, age, socioeconomic level, and marital status.

#### Reason for admission to the institution

Specify the reason why the individual was admitted. If the user committed a crime, it is ascertained whether he/she was under the influence of a substance. It is also noted whether any substance was used 6 h prior to admission to the institution, specifying type and dose.

#### Associated problems before and after substance use

According to the user’s perception, problems associated with substance use, both before and after it, are identified, as well as the problem which the user cites as being most important.

#### Substance use

The following are the types of substances investigated:

Medical substances: amphetamines and stimulants, sedatives and tranquilizers, other opiates, and other medical substances.

Non-medical substances: hallucinogens, cocaine, heroin, inhalants, marijuana, and other non-medical substances.

##### Socially accepted substances: alcohol and tobacco

The following aspects are evaluated for each substance: lifetime prevalence, use in the past year, frequency of use in the past month, year and age at onset of use, routes of administration of each substance, and the specific name the latter is given by the user.

A fillable form is delivered twice a year to institutions that attend users for their care and treatment. This form is completed by health personnel previously trained by researchers from the National Institute of Psychiatry. The person in charge of giving the information handles the user’s file. Users voluntarily attend these institutions to receive attention. The schedule is completed once the user’s evaluation is completed. It is not filled out directly by the user, but by the health personnel responsible for the treatment.

Participating institutions provide biannual data on the population they summon during the application period. Each evaluation is cross-sectional, in other words, it is undertaken at a given time and provides an account of the state of the phenomenon at that point in its history. Once the evaluation period is over, information on the cases is compiled by the National Institute of Psychiatry for processing and analysis.

### HIV/AIDS variables

Data reported by the Epidemiological Surveillance System [15] were used. The surveillance system works as follows. Physicians have the obligation to report all HIV and AIDS cases. Every time a new case is detected and confirmed by the Western blot test, physicians collect basic demographic information on the patient, including his/her name, age, occupation, and educational attainment. Some behavioral variables are also recorded, such as the type of sexual relations in which respondents engage, their experience with commercial sex, history of blood transfusions, and use of injected drugs. This information is received by the Ministry of Health of each state before submitting it to the central offices in Mexico City. Follow-up of these subjects is required at six-month intervals for AIDS cases and 12-month intervals for seropositive individuals.

### Analysis

This is a descriptive study which presents the frequencies and percentages of the drugs and HIV/aids cases being reported and registered by the instruments mentioned above.

## Results

The substances considered in the analysis were all those used intravenously: amphetamines, cocaine, heroin, opiates, hallucinogens, sedatives, and other non-medical drugs.

Table 1 shows the user’s profile for each type of substance. The highest proportion of consumers are men, most of whom are married with a medium socioeconomic status. Most of them did not report having any occupation, except for sedative users, who were mostly (27.4%) employees or vendors.

**Table 1:** PWID profile(*N* = 740)

	Amphetamines	Cocaine	Heroine	Opiates	Hallucinogens	Sedatives	Other non medical drugs
	*n* = 18 (2.4%)	n = 293 (38.2%)	*n* = 184 (24.9%)	*n* = 90 (12.6%)	*n* = 39 (5.3%)	*n* = 63 (8.5%)	*n* = 53 (7.2%)
Sex							
Male	83.3	84.3	83.6	81.1	84.6	69.8	79.2
Female	16.7	15.7	16.4	18.9	15.4	30.2	20.8
Civil Status							
Married	–	91.8	84.7	86.7	79.5	92.1	88.7
Divorced	–	–	–	–	–	–	–
Separated	–	–	–	–	–	7.9	–
Single	100	7.2	13.7	13.3	17.9	–	11.3
Common law	–	1.0	1.6	–	2.6	–	–
Socioeconomic level							
Low	100.0	14.7	15.8	21.1	23.1	12.5	11.3
Medium	–	85.3	84.2	78.9	76.9	87.3	88.7
Education level							
S/E	–	2.1	1.7	1.1	–	3.3	–
Primary school incomplete	–	7.3	5.5	4.5	5.1	1.6	3.8
Primary complete	5.6	7.7	7.7	6.7	5.1	8.2	3.8
Secondary incomplete	27.8	19.9	23.8	28.1	10.3	26.2	7.5
Secondary complete	22.2	18.8	17.1	21.3	12.8	24.6	20.8
Technical incomplete	–	1.7	2.8	–	5.1	–	–
Technical complete	–	5.2	3.3	5.6	–	4.9	1.9
High school incomplete	22.2	25.1	18.2	12.4	46.2	14.8	30.2
High school complete	16.7	4.9	12.2	6.7	12.8	6.6	18.9
University studies incomplete	5.6	5.2	5.5	7.9	2.6	1.6	5.7
University complete	–	1.7	2.2	5.6	–	6.6	7.5
Postgraduate incomplete	–	–	–	–	–	–	–
Postgraduate complete	–	.3	–	–	–	1.6	–
Occupation							
Housewife	–	2.5	1.7	4.7	–	6.5	3.8
Employee or retailers	50	23.2	16	–	7.9	27.4	18.9
Student	18.8	12.9	10.9	5.8	34.2	14.5	18.9
Professionist	–	2.5	0.6	3.5	–	1.6	1.9
No occupation	12.5	29.3	41.2	36.0	36.8	24.2	24.5
Underemployed, casual laborer	18.7	29.7	29.7	26.7	21.0	25.8	32.1

Table 2 shows the age groups and the type of users. The most frequent age group was between the ages of 15 and 19, followed by those aged 20 to 24 in all cases. The year of onset of use varies, with heroin and opiate user groups reporting the longest lengths of use with 10 years or more.

**Table 2:** PWID profile (*N* = 740)

	Amphetamines *n* = 18 (2.4%)	Cocaine *n* = 293 (38.2%)	Heroine *n* = 184 (24.9%)	Opiates *n* = 90 (12.6%)	Hallucinogens *n* = 39 (5.3%)	Sedatives *n* = 63 (8.5%)	Other non medical drugs *n* = 53 (7.2%)
Age							
<12	–	1.0	0.6	2.4	–	–	–
12–14	5.6	9.0	10.9	4.7	7.8	14.5	12.6
15–19	50.1	39.3	43.1	32.5	71.9	43.6	59.3
20–24	27.8	26.9	26.4	29.1	15.5	24.1	22.3
25–29	16.7	13.5	12.7	17.2	5.2	14.4	5.8
> 30	–	9.7	6.3	14.1	–	3.2	–
Year of onset							
<1969	–	–	1.8	1.1	–	–	–
70–72	–	–	1.1	2.3	2.6	–	–
73–75	–	0.6	2.3	1.1	–	–	–
76–78	–	1.6	3.4	2.3	–	3.2	–
79–81	–	8.0	2.2	2.2	–	3.2	–
82–84	5.6	4.1	3.4	4.5	5.2	4.8	–
85–87	5.6	4.1	3.9	6.7	2.6	6.4	–
88–90	–	6.6	8.5	9.1	2.6	4.8	–
91–93	–	16.2	11.2	13.7	5.1	9.6	4.0
94–96	–	18.8	10.2	6.7	2.6	3.2	7.8
97–99	50.0	22.6	10.1	16.0	2.6	11.9	5.9
00–02	–	5.8	14.4	6.2	5.1	17.9	19.6
03–05	22.2	3.8	5.7	5.6	5.1	8.0	19.6
06–08	5.6	3.1	7.3	8.0	7.8	1.6	7.9
09–11	5.6	2.4	10.1	9.0	12.8	9.6	5.9
12–14	5.6	1.7	4.4	4.4	33.4	16.1	29.4
15–17	–	0.6	–	1.1	12.3	–	–
Type of user							
Ocassional	–	8.2	10.3	–	–	6.8	11.6
Mild	27.8	42.7	40.2	27.8	45.8	36.4	41.9
Moderate	50.0	18.5	22.7	33.3	29.2	27.3	23.3
High	22.2	30.6	26.8	38.9	25.0	29.5	23.3
Ocassional consumption in the last year, but not in the last month. Mild consumption in the last month, from 1 to 5 days. Moderate consumption in the last month, from 6 to 19 days. High consumption in the last month for 20 days or more.							
Mean number of drug per user	3.00	4.0	4.0	4.0	4.0	4.0	4.0
Average number of problems before and after consumption	5.0/5.0	5.0/5.0	5.0/5.0	3.5/3.0	4.5/5.0	5.0/5.0	5.0/5.0

By type of use, those who used cocaine, heroin, hallucinogens, sedative, and other non-medical drugs were light users. In the case of amphetamines, moderate use was reported, with the group of opioid users reporting the highest rates of use.

**Fig. 1:**
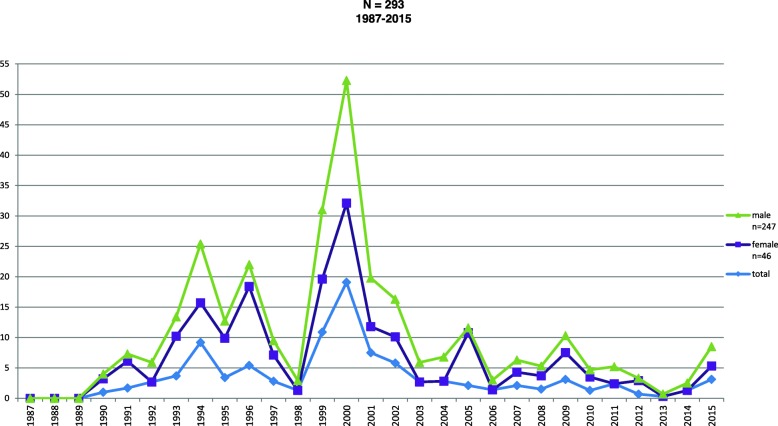
Cocaine PWID trends by sex

Figure 1 shows trends in cocaine from 1997 to 2015. This substance had the highest number of users, with a total of *N* = 293. The graph shows how, in 2000, the use of this substance showed a significant increase of up to 50%

**Fig. 2:**
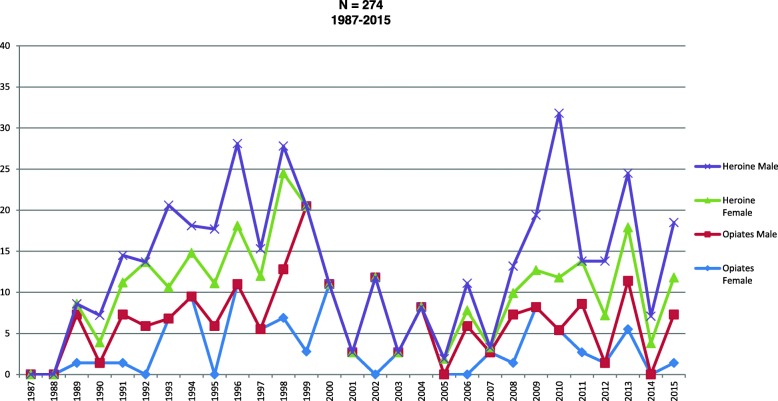
Heroine and opiates PWID trends by sex

Figure 2 present opiate and heroin use cases, which went from being almost non-existent in 1987 to 13% in 1994. This graph provides evidence of the change in the number of users over the years, with the largest increases being recorded in 1996 (16.5%), 1999 (17%), and subsequently in 2010 (13%). Opioids, morphine, nalbuphine, codeine, and nubain are among the most widely used opioids

**Fig. 3:**
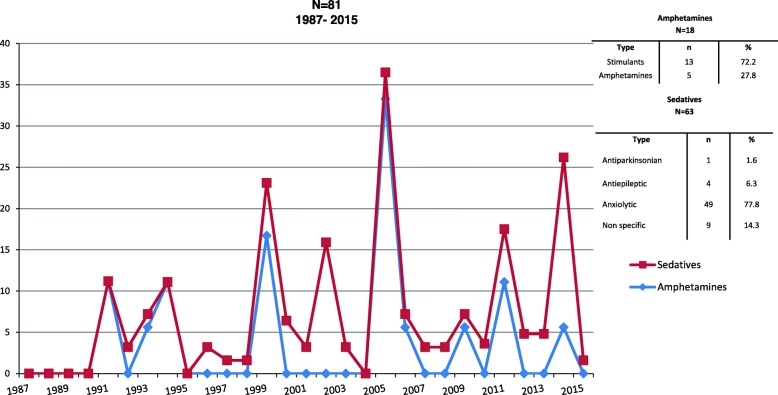
Amphetamines and sedatives PWID

Figure 3 expose the increase in amphetamine and sedative users, in parallel, in two periods between 2000 and 2006, and subsequently in 2014

**Fig. 4:**
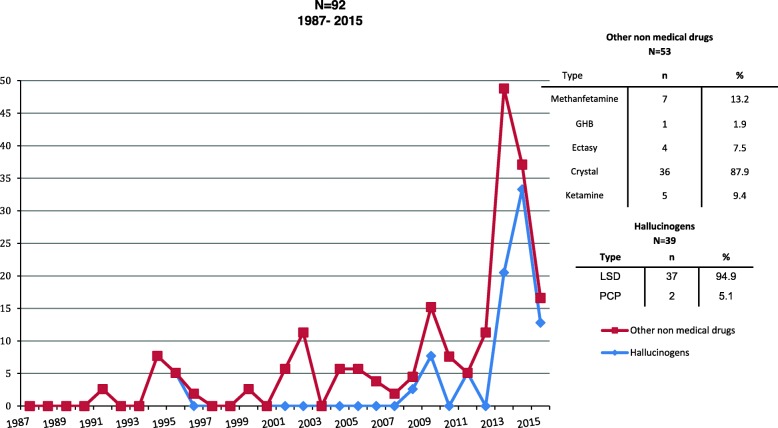
Other non-medical drugs and hallucinogens PWID

Figure 4 which remained low for 25 years, at less than 10% and fluctuating between 0 and 5%, until 2013, when use increased to 50%. The most commonly used non-medical drugs are crystal, GHB, ecstasy, and ketamine, while LSD and PCP are the most popular hallucinogens

**Fig. 5:**
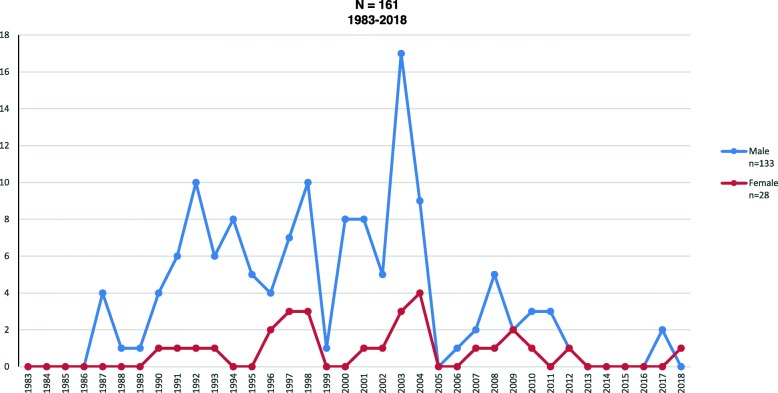
PWID cases with aids, SUIVE/DGE/SS. Epidemiological Surveillance System of hiv/aids. Preliminar information second trimester 2018. National Center for the Prevention and control of hiv/aids. Direction of Integral Attention

Table 3 all users reported a problematic drug use and had on average five problems (including personal, economic, and social issues). Family problems were the most common for all types of users, excepting for those who used amphetamines, who pointed out that mental or nervous problems were the more important.

**Fig. 6:**
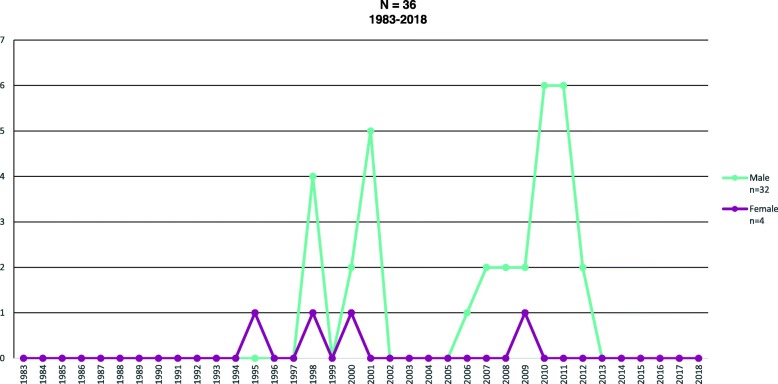
PWID cases with HIV, SUIVE/DGE/SS. Epidemiological Surveillance System of hiv/aids. Preliminar information second trimester 2018. National Center for the Prevention and control of hiv/aids. Direction of Integral Attention

**Table 3:** Associated Problems Percentages (*N* = 740)

	Amphetamines *n* = 18		Cocaine *n* = 293		Heroine *n* = 184		Opiates *n* = 90		Hallucinogens *n* = 39		Sedatives *n* = 63		Other non Medical drugs *n* = 53	
	Before Use	After	Before Use	After	Before Use	After	Before Use	After	Before Use	After	Before Use	After	Before Use	After
Academic	16.7	24.4	12.5	42.9	15.6	53.0	13.5	43.9	7.7	19.4	14.8	17.9	17.0	31.7
Economic	5.9	38.9	15.4	41.8	11.9	47.2	8.2	41.9	2.6	15.4	10.0	20.0	5.8	17.3
Familiar	35.3	53.8	34.8	63.1^*^	40.3	75.7^*^	25.6	72.0^*^	2.6	38.9^*^	16.7	47.5^*^	26.4	48.3^*^
Work	11.1	40.0	12.9	41.0	13.6	54.0	11.8	45.0	2.6	21.9	6.6	26.5	3.8	13.0
Legal Issues	5.6	37.5	8.7	44.0	8.4	57.2	5.7	35.7	5.1	25.0	0	12.5	1.9	15.2
Mental- Nervous	27.8	61.1^*^	30.3	57.6	45.8	55.6	28.1	48.8	15.4	15.4	6.7	30.0	9.6	32.1
Physical	5.9	41.2	10.0	47.8	13.1	50.9	10.3	48.7	0.0	24.0	8.2	30.0	7.7	27.0
Psychological	38.9	43.8	33.7	42.7	47.2	60.1	33.3	47.4	33.3	24.0	28.8	35.4	9.4	33.3
Sexual	38.9	38.9	27.6	34.0	40.1	44.8	25.8	36.0	33.3	33.3	25.4	28.8	3.8	9.4
Social	11.1	38.9	12.4	41.0	20.0	54.3	8.2	38.6	18.9	18.8	20.0	22.2	5.7	29.8

Academic: Learning problems, Bullyng, behavioral problems

Economic problems: low income, spend money on drugs

Familiar problems: family rupture, domestic violence, family consumption

Work problems: job instability, Job’s issues

Legal issues: robberies car accidents, drug sales

Mental-nervous: Anxiety disorders, depressions, attention deficit/hyperactivity disorder, schizophrenia

Physical problems: cardiovascular problems, respiratory problems, astenia

Psychological problems: low self-esteem, suicide attempt, Low tolerance to frustration

Sexual problems: sexual abuse, Increased sex drive, Lack of sexual interest

Social problems: antisocial behaviors, isolation, environment influence

*Highest percentage of problems in every type of drug

Table 4 users consume on average five drugs, the most frequent combination being heroin and cocaine, known colloquially by users as speedball. Hallucinogens were the substances more widely combined with cocaine y amphetamines.

**Table 4:** injectable Substances combination percentages

Injectable Substances *N* = 740	Injectable Substances Use Combinations	%
Amphetamines f = 18	Sedatives (1)	16%
	Hallucinogens (2)	
Opiates f = 90	Hallucinogens (1)	26.4%
	Heroine (7)	
	Other non Medical drugs (1)	
	Sedatives (1)	
	Cocaine (14)	
Sedatives f = 63	Amphetamines (2)	12.7%
	Hallucinogens (1)	
	Heroine (4)	
	Opiates (1)	
Hallucinogens f = 39	Amphetamines (2)	23.6%
	Heroine (1)	
	Other non Medical drugs (1)	
	Cocaine (3)	
	Opiates (1)	
	Sedatives (1)	
Cocaine f = 293	Heroine (17)	12.3%
	Other non Medical drugs (2)	
	Opiates (14)	
	Hallucinogens (3)	
Heroine F = 184	Other non Medical drugs (3)	17.4%
	Opiates (7)	
	Sedatives (4)	
	Hallucinogens (1)	
	Cocaine (17)	
Other non Medical drugs f = 53	Opiates (1)	13.2%
	Hallucinogens (1)	
	Cocaine (2)	
	Heroine (3)	

Figure 5 reach a peak in 2003 with 161 cases followed by an abrupt decrease in registered cases, being mostly males

Figure 6 shows, as in the previous case, an increase in cases in 2002, but unlike the reduction in AIDS cases, here there was a subtle increase between 2005 and 2013 in HIV cases

## Discussion

From a historical point of view, the increase observed in the results coincides with domestic and world political situations that caused an upturn in the use of certain substances over the years [16]. In Mexico, President Salinas’s administration (1988–1994) witnessed the start of cocaine use, which peaked during the government of his successor, President Zedillo (1994–2000, 17). According to Gaussens [2], Mexican drugs exports in millions of dollars increased to 7325 during Salinas’ government, accounting for 1.9% of the gross domestic product. In previous administrations, the figure was significantly lower.

As a result of this increase in use, the 1990s also saw a greater interest in studying cocaine use -- through various methodological approaches -- in Mexico City and its link with the risk of disease transmission by parenteral means [17].

The issue of heroine and opiates use in Mexico is not a new phenomenon. Between 1976 and 1982, records from the Centros de Integración Juvenil reported that two of every 100 patients consumed heroine. However, it is only during the 1990s when an increase in the number of users of this drug can be demonstrated, which went from 1.8% in 1990 up to 4.0% in 2000. This came to be because the Epidemiological Surveillance System on Addictions of the Ministry of Health started to report on a periodical basis patient looking for treatment. From all the substances included here, it can be seen that heroine and cocaine use, even when it presents ups and downs and is growing in women, is the use which has remained more stable and its users are not occasional but moderate to high.

Regarding the use of amphetamines, we can state that the increase visible in 2005, when the highest number of cases is recorded, runs in parallel with a historical period of time when great amounts of ephedrine and pseudo-ephedrine were produced illegally. Later on, they were forbidden to be imported into Mexico. These substances are essential ingredients to produce amphetamines. According to data from General Attorney of the Republic (PGR) and the Drug Enforcement Administration (DEA), more than 30 individuals linked with the businessman Zhenil Ye Gon – associated with the Sinaloa Cartel and Los Zetas – were arrested and 207 million dollars were confiscated. So far, the case is not judicially solved [18].

The increase of other non-medical drug use is a phenomenon that has emerged more recently, especially crystal. Although only 18 cases have been registered in this reporting system, methamphetamine is one of the most harmful drugs and the number of experimental users may be high [19]. In Mexico, Sonora, Sinaloa, and Baja California are the states where its use is the highest. For Mexico City, the data mark the beginning of the 2000s as the period of time when there was an upturn in use and again in 2014. According to Martínez [20], methamphetamine (speed, crystal, ice, shabu, meth, or chalk) is a stimulant-type substance that acts by releasing dopamine, serotonin, and noradrenaline. It inhibits the recapture of neurotransmitters of the postsynaptic neuron, in addition to stimulating the reward mesocorticolimbic system, which is susceptible to abuse and the development of dependence. According to the National Institute of Drug Abuse [21], methamphetamine is a highly dangerous drug, recognized as the most toxic and addictive one, because it encourages ten times more than normal dopamine production and causes extensive brain damage. It is long-acting and requires up to 12 h to be eliminated, not to speak of its psychostimulant effects. It causes euphoria, a feeling of happiness, increases the attention span and libido, and reduces fatigue. Over time, the user loses the ability to produce dopamine naturally and may display symptoms resembling those of Parkinson’s disease. It is also important to emphasize that these injectable substances can be combined with each other or become more complex by being added to other substances, such as alcohol, causing more severe damages to health and increasing the possibility of death due to overdoses [22].

In comparing these results with those from other drugs information sources and concerning Mexico City in particular, we have found the following:

According to information from the 2019 Mexican Observatory of Drugs [23], Mexico City occupies the tenth place (out of 33 states) as to the demand of treatment from users of injectable drugs. From a total of 2647 cases on a national level who looked for help in 2016, only 26 men and six women lived in Mexico City and its metropolitan area. In decreasing order, the states with more demand for help were Sonora, Chihuahua, Baja California, Guerrero, Michoacan, Jalisco, Guanajuato, Morelos, and Puebla.

Regarding emergency admissions for the consumption of any type of drug in 2017 and according to the CIE-10, in Mexico City there were 14,337, which places it above the national median (X 0 1351). From these admissions, 4.7% were from F14: cocaine use, and 0.98, F11: opiates use [23].

Sticking to the same source and according to the CIE-10, there were 144 cases of hospital discharges from mental disorders and from opiate-related behaviors. From them, the first place was Sonora with 74 cases and then Mexico City with 28.

All this lets us see that, while the number of cases is not the highest in the country, some indicators point out that these are on the rise.

In relation to HIV/AIDS we can say that the high amount of AIDS prevention campaigns carried out since the beginning of the epidemic in our country were not directed to a specific population selected for their sexual orientation. Instead, they were fundamentally directed to inform people and sensitize the entire population on the subject, particularly young people and their main references. The groups of the population targeted by the first focused strategies were men who have sex with men, workers and commercial sex workers and street children. Beginning in 1990, strategies aimed at other groups were initiated, with injecting drug users, prisoners, indigenous people, mobile populations, and rural communities being the ones who joined later. In 1997, the first results of a study on injecting drug users in the city of Tijuana were published and CENSIDA decided to develop a risk reduction prevention strategy for this group [24]. In 2016, the *Guide for the Use of Methadone in Adult Users with HIV Dependent on Intravenous Heroin* was published [25].

In Mexico, the use of antiretroviral drugs began to be used in 1997, but it was in 2003 when it became a public health policy of free and universal access, in such a way that it was possible to incorporate treatment that did not have social security. As a result, by the end of 2012 a total of 84,146 people was receiving antiretroviral treatment. In 2013, HIV/AIDS ranked fifteenth as the cause of death in the general population, with a total of 4965 deaths and a rate of 4.19 per 100,000 inhabitants. This means that 13–14 people died every day from HIV/AIDS in Mexico. The mortality rate in men has been about five times higher than that recorded for women [24].

Some of the limitations of this study derive from the ever-changing number of institutions involved, a fact which may be explained by the publication of Norma 028 [26]. Nom-028 is the legislation for the Integral Attention of Addictions. One of its guidelines is to guarantee, through the Methadone Clinics Program, that methadone substitution therapy is available to those heroine use patients. Guidelines to follow are related licenses and permits (SSA, COFEPRIS) to handle the medication inside the clinics’ premises (storage, custody, prescription, administration, dosages register), the patient’s treatment, the necessary medical personnel (psychiatrist, general physician, clinical psychologist, etc.), the premises features (reception, outpatient consultation unit, medical office, etc.), among others [26].l NOM-028-SSA2–2009 was drawn up in response to the need to guarantee quality care in service provision to reduce the incidence and prevalence of the use of illegal substances or those without medical prescription.

As a result of the observation and evaluation of compliance with the norm, some centers such as non-governmental organizations operated in a limited way and once they had been evaluated, were forced to close until they complied with government observations and some never re-opened. This process was necessary to guarantee prevention, detection and treatment operations. The SRID System observed that the number of collaborating institutions decreased and that in other cases, they presented more problems in order to continue filling out the form.

It came into force as of its publication in September 2000 and continues to operate under the supervision of the Institute for the Care and Prevention of Addictions in Mexico City. The new panorama for addressing public health problem requires health professionals committed to compliance with standards. The change of authorities within the institutions as well as in the structures has been a constant, and in some cases, has caused the temporary or definitive interruption of the use of the SRID form. It is our hypothesis that they were overwhelmed by the number of requests for care, regulation and compliance with professional training and/or certification of person.

In 2016, the System completed 30 years of uninterrupted operation, registering between 20 and 40 collaborating institutions at various times. However, it is only applied twice a year and law enforcement institutions have ceased to participate. Nowadays, the System has 18 health, Non-Governmental Organizations and Law Enforcement institutions, which collaborate freely and autonomously in the application of the form twice a year.

Another limitation is the fact that institutions providing treatment to high socioeconomic level patients were not represented. Starting 2019, some private associations have decided to collaborate.

## Conclusions

It is plausible to think that in the coming years, there will be an increase in the number of cases of persons who inject drugs due to the high production and availability of heroin in Mexico [27] and the emergence of the use of fentanyl as indicated by the ethnographic research in Mexico City and the deaths linked to its consumption [22]. The latest reports of the SRID, analyzed in 2018, documented at least five cases of fentanyl users. This information is not still completed for its publication.

Both epidemiological surveillance systems, the SRID and the HIV/AIDS should be used simultaneously and include their application in other places where users are common, such as preventive prisons and Social Readaptation Centers where the people with the highest number of risk behaviors in Mexico City are located. If we consider that justice institutions are often a revolving door in this population group, in other words, they enter and leave the facilities quickly, only to re-enter in the short term, this is where there is an opportunity for intervention with harm reduction strategies [25].

A greater number of projects are probably necessary of a qualitative nature, to answer the question if only the greater availability of the substance is the response to the increase in the phenomenon. Harm reduction strategies are necessary to avoid the deaths of users.

## Data Availability

Data used in this article is available upon request to the corresponding author.

## Abbreviations

AIDS: Acquired Immune Deficiency Syndrome
CENSIDA: National Center for the Prevention and Control of HIV/AIDS
HIV: Human immunodeficiency virus
PWID: people who inject drugs
SRID: Drug Information Reporting System
USD: United States dollars
